# Evaluation of the Role of Enteral Nutrition in Managing Patients with Diabetes: A Systematic Review

**DOI:** 10.3390/nu6115142

**Published:** 2014-11-18

**Authors:** Omorogieva Ojo, Joanne Brooke

**Affiliations:** Faculty of Education and Health, University of Greenwich, Avery Hill Road, Avery Hill Campus, London, SE9 2UG, UK; E-Mail: J.M.Brooke@greenwich.ac.uk

**Keywords:** enteral nutrition, diabetes, diabetes specific formula, standard formula, hyperglycaemia, glycaemic index

## Abstract

The aim of this systematic review is to evaluate the role of enteral nutrition in managing patients with diabetes on enteral feed. The prevalence of diabetes is on the increase in the UK and globally partly due to lack of physical activities, poor dietary regimes and genetic susceptibility. The development of diabetes often leads to complications such as stroke, which may require enteral nutritional support. The provision of enteral feeds comes with its complications including hyperglycaemia which if not managed can have profound consequences for the patients in terms of clinical outcomes. Therefore, it is essential to develop strategies for managing patients with diabetes on enteral feed with respect to the type and composition of the feed. This is a systematic review of published peer reviewed articles. EBSCOhost Research, PubMed and SwetsWise databases were searched. Reference lists of identified articles were reviewed. Randomised controlled trials comparing enteral nutrition diabetes specific formulas with standard formulas were included. The studies which compared diabetes specific formulas (DSF) with standard formulas showed that DSF was more effective in controlling glucose profiles including postprandial glucose, HbA1c and insulinemic response. The use of DSF appears to be effective in managing patients with diabetes on enteral feed compared with standard feed.

## 1. Introduction

The prevalence of diabetes and the cost to the National Health Service (NHS) have been on the increase in the UK [1,2]. Various factors including the failure to maintain a healthy lifestyle such as regular physical activity and healthy dietary regimes, and genetic susceptibility have been ascribed as possible reasons for the high incidence of the condition [3,4]. The manifestation of diabetes comes with various complications such as cerebrovascular accident, which may result in dysphagia, often requiring nutritional support [5,6]. This is especially evident in patients with diabetes who may be unable to maintain their nutritional requirements through the use of oral dietary intake alone and thus may require enteral feed. In addition, their involvement in physical activity that would ensure the maintenance of normal glucose levels may be compromised due to their neurological conditions and poor mobility [7,8]. Therefore, managing a person with diabetes on enteral nutrition could present some difficulties for the multidisciplinary healthcare professional (HCP) team if there are no effective strategies for managing the enteral feed [9]. There could be increased risk of the patient with diabetes on enteral feed developing hyperglycaemia or hypoglycaemia, which can result in potentially poorer clinical outcomes [10,11,12].

In a study by Ojo [13], comparison of patients on home enteral tube feeding (HETF) with the Quality and Outcome Framework (QOF) data revealed that diabetes prevalence in people on HEFT in Lambeth, Lewisham and Southwark primary care trusts in the UK was significantly higher (7.78%) than in the general population (3.63%) not on enteral nutrition and living in the same area. Ojo [13] showed that more patients who have diabetes are now requiring enteral nutrition support.

The role of enteral nutrition in patients with diabetes is to provide the required macro- and micro-nutrients including energy, protein, vitamins and minerals in part or whole in order to reduce the risk of malnutrition in these patients [14]. However, due to the nature of the different food formulas, the risk of hyperglycemia could be a major challenge in these patients and newly diagnosed hyperglycaemia could be considered an independent prognostic factor of mortality in patients with enteral feeding [15,16]. Hyperglycaemia can have profound impacts on a range of patients with diabetes including those hospitalised, such as patients with diabetic ulcers or undergoing limb amputations. Hyperglycemia may impact on wound healing, time spent in hospital and lead to complications including diabetic ketoacidosis and hyperosmolar non-ketotic state [17,18]. Therefore, there is the need to ensure adequate management of patients with diabetes, especially those on enteral feed because of the effects on blood glucose levels. The cost of major complications resulting from hyperglycaemia to the UK economy has been estimated to be between £872 for blindness in one eye to £8459 for amputation per patient, although the total cost of type 2 diabetes to the UK economy appears difficult to evaluate [19]. However, in 2007 estimates of 7%–12% of the total NHS budget, which could be £2.8 billion associated cost for the UK has been reported [1,20].

Nutritional requirements of patients with diabetes on enteral nutrition are met with the use of standard enteral feeds or diabetes specific feeds. Diabetes specific formulas contain specific ingredients that often include fructose and a large amount of monounsaturated fatty acids, which are aimed at controlling postprandial glucose [21,22]. The effects of these feeds in maintaining the nutritional requirements and physiological state of patients with diabetes continue to generate debate and attract the interest of researchers. A scoping exercise of the literature revealed two systematic reviews on the role of enteral nutritional support and the use of diabetes specific formulas for patients with diabetes carried out at various times with different findings [19]. A systematic review and meta-analysis of enteral nutritional support and use of diabetes specific formulas conducted by Elia *et al.* [19] aimed to determine the benefits of nutritional support in patients with types 1 and 2 diabetes. It compared the use of nutritional support with routine care, and standard formulas with diabetes formulas. Although the study concluded that the use of diabetes specific formulas (DSF) as oral nutrition supplements and tube feeds improve blood glucose levels when compared with standard formulas, controversies still surround the use of DSF. In particular, there are clinical interests in establishing the safety and tolerance of relatively high levels of fat and fructose in patients with underlying dysmotility disorders such as irritable bowel syndrome and with respect to lipid metabolism and lactic acidosis [22]. Since the study by Elia *et al.* [19], a number of randomised controlled trials based on patients with diabetes on enteral nutrition have been published. In addition, the American Society of Parenteral and Enteral Nutrition (ASPEN) Clinical Guidelines: nutrition support of adult patients with hyperglycaemia which was developed in order to provide the desired blood glucose goal in hospitalized patients receiving nutritional support could not recommend whether diabetes specific formulas can be used for hospitalised adult patients with hyperglycaemia [23].

The ASPEN Clinical Guidelines recommendation for the use of diabetes specific formula was based on only two studies published in 2003 and 2005. It was therefore not surprising that the ASPEN guideline recommended that further research was required in the use of diabetic specific formulas [23]. According to Cheng [24], two strategies for managing hyperglycaemia of enteral feeding are adjustment of the enteral feed carbohydrate content and pharmacological therapy to lower glucose levels although the current review is focused on the former.

Drawing from the above reviews and guidelines, the need to examine the role of standard *versus* diabetes specific formulas has become pertinent. Therefore, the aim of the present study is to carry out a systematic review of the role of enteral nutrition in supporting patients with diabetes. However, the use of insulin and oral hypoglycaemic agents was not examined in this review.

The objective is as follows:

- To examine the effects of standard and diabetes specific enteral formulas in the management of diabetes.

The research question is:

- Is diabetes specific formula more effective than standard formulas in managing patients with diabetes on enteral feed?

## 2. Experimental Section

A systematic review was carried out based on published guidelines [25,26]. This involved a literature search of articles of interest relating to the use of enteral nutrition in diabetes management, including a general scoping of the data bases which found only two systematic reviews that were relevant to the population and intervention of interest. A search of the Cochraine library and databases of abstracts of reviews and effects found one article by Elia *et al.* [19], which was published in 2005. A further search of SwetsWise and EBSCO host databases found the ASPEN guidelines; nutrition support of adult patients with hyperglycaemia [23]. Although this guideline was published in 2013, it derived most of its limited evidence with respect to the question on the use of diabetes specific formulas in adult hospitalised patients with hyperglycaemia from studies published between 2003 and 2005.

The research question was defined into the component parts; the Population (P), the Interventions (I), Comparative interventions (C) and Outcomes (O) based on PICO framework [25]. Table 1 shows the results of the various searches. The databases searched included EBSCO Host/Health Sciences Research databases (encompassing Academic search premier, Medline, Psychology and Behavioural sciences collection, PSYCINFO, SPORTDISCUSS and Cumulative Index to Nursing and Allied Health Literature (CINAHL) Plus) and SwetsWise. The reference list of relevant systematic reviews and articles were checked in order to identify studies that could be useful to the present review.

**Table 1:** Literature search strategy.

Database	Dates Covered	Date Searched	Hits	Search Terms
EBSCO Host (Health Sciences Research Databases)	2005–2014	04.06.14	469,184	Diabetes
EBSCO Host (Health Sciences Research Databases)	2005–2014	04.06.14	323	Diabetes and Enteral Nutrition
EBSCO Host (Health Sciences Research Databases)	2005–2014	04.06.14	13	Diabetes and Enteral Nutrition and Diabetes Specific formula
EBSCO Host (Health Sciences Research Databases)	2005–2014	04.06.14	2	Diabetes and Enteral Nutrition and Standard Feed
EBSCO Host (Health Sciences Research Databases)	2005–2014	04.06.14	1	Diabetes and Enteral Nutrition and Glycated Haemoglobin
EBSCO Host (Health Sciences Research Databases)	2005–2014	04.06.14	4	Diabetes and Enteral Nutrition and Fasting Blood Glucose
EBSCO Host (Health Sciences Research Databases)	2005–2014	04.06.14	28	Diabetes and Enteral Nutrition and Hyperglycaemia
EBSCO Host (Health Sciences Research Databases)	2005–2014	04.06.14	11	Diabetes and Enteral Nutrition and Hypoglycaemia
SwetsWise	2005–2014	04.06.14	46	Diabetes and Enteral Nutrition
SwetsWise	2005–2014	04.06.14	47	Diabetes and Standard feed
SwetsWise	2005–2014	04.06.14	73	Diabetes and Diabetes Specific feed

### 2.1. Inclusion and Exclusion Criteria

Only randomised controlled trials were selected for inclusion in the current review.

The participants in the studies included in the systematic review were adult males and females who had diabetes and were on enteral nutrition while the interventions were standard enteral feed and DSF.

The outcome measures included in the search were; HbA1c, fasting blood glucose, hyperglycaemia and hypoglycaemia.

In terms of the years of publication, searches were conducted between 2005 and 2014 while only studies written in English language were included. Studies that did not meet the above inclusion criteria were excluded from the study.

### 2.2. Data Analysis

Based on the criteria outlined for exclusion and inclusion of various studies, 469,184 articles were initially available. This number was further reduced to 323 articles with the inclusion of enteral nutrition as part of the search term (Table 1). Of these articles, five articles that met the requirements for selection were included in the review (Table 2).

## 3. Results

All the studies included in the systematic review involved patients who had type 2 diabetes and these were clearly specified in the method section in the respective studies. While three studies compared DSF with standard formula [21,27,28], one study [29] compared slowly digested carbohydrate formula with DSF and standard formula (Table 2). The final study compared diabetes specific enteral formula with 49.95% calories provided by fat and diabetes specific enteral formula with 34% calories provided by fat [30].

In the study by Ceriello *et al.* [21], glucose profiles were significantly better after administering DSF compared with standard formula. In addition, diabetes specific formula significantly improved postprandial glucose compared with the standard, fibre-containing formula.

With respect to the study by Vaisman *et al.* [28], HbA1c decreased over time in the diabetes specific diet group and increased in the standard feed group. Similarly, in the randomised double-blinded study evaluating the postprandial glycaemic and insulinemic response conducted by Alish *et al.* [27], differences in adjusted peak plasma glucose levels were significantly lower in DSF compared with standard formulas (STF) (*p* < 0.001). In addition, differences in adjusted peak insulin levels were significantly lower in DSF compared with STF (*p* = 0.017). In other words, the use of DSF produced significantly lower postprandial and insulinemic response.

Voss [29] revealed that adjusted glucose concentrations were significantly higher at all points after feeding the STF compared with the slowly digested carbohydrate formula (SDC) or DSF (*p* < 0.001). In addition, adjusted plasma glucose concentrations for the SDC were observed to be significantly lower than those for the DSF from 30 to 120 min (*p* < 0.05). In relation to the adjusted insulin responses, these were significantly higher for the STF compared with the SDC at each time point, while the SDC had a significantly lower insulinemic response compared with the DSF at 90 and 120 min (*p* < 0.05) [29]. Finally, the adjusted results for postprandial glucagon-like polypeptide-1 (GLP-1) levels at 30 and 60 min in this study were significantly lower for the STF compared with SDC (*p* < 0.05), but not different from the DSF [29].

In the study by De Luis *et al.* [30] which compared diabetes specific enteral supplement with 49.95% calories provided by fat (Gp 1) and diabetes specific enteral supplement with 34% calories provided by fat (Gp 2), a significant decrease of glucose and HbA1c was observed in Gp 1 compared with Gp 2.

**Table 2:** Summary of studies reviewed.

Citation	Study Type	Population and Sample Size	Age (Years)	Intervention	Outcomes	Remarks
Ceriello A *et al.*, 2009 [21]	Randomized Controlled Study	12	67.2 ± 1.3	Diabetes-Specific Formula (DSF) Standard Formula (STF)	Fasting Glucose (mmol/L) = 7.9 ± 0.45	Results are expressed as mean ± SEM
					24 h Glucose Concentrate (mmol/L-SEM) = 8.7 ± 0.5	
					Day time = 9.4 ± 0.6	
					Fasting Glucose (mmol/L) = 7.6 ± 0.37	
					24 h Glucose Conc. (mmol/L-SEM) = 9.6 ± 0.6	
					Day time = 10.7 ± 0.6	
Voss AC, 2008 [29]	Randomized Controlled Study	48	56 ± 1.4 (SEM)	Slowly Digested Carbohydrate Formula (SDC) DSF STF	The positive area under the curve for glucose and insulin with STF was higher (*p* < 0.001) compared with the SDC and DSF. The adjusted Glucagon-like peptide-1 (GLP-1) concentration at 60 min. was higher for the SDC compared with the DSF and STF (*p* < 0.05).	Results are expressed as mean ± SEM
De Luis *et al.*, 2008 [30]	Randomized Clinical Trial	16 14	74.6 ± 7.1 77.1 ± 8.7	Diabetes Specific Enteral Formula (49.95% of Calories provided by fat, Gp 1) Diabetes Specific Enteral Formula (34% of Calories provided by fat, Gp 2)	Baseline Glucose mg/dL = 119.8 ± 42 10 weeks = 95.1 ± 16.8 HbA1c% = 8.2 ± 2.8 at baseline, 5.8 ± 0.7% at 10 weeks	Results are expressed as mean ± SD
					Baseline Glucose mg/dL = 122.4 ± 22.8 10 weeks = 130.6 ± 41.4	
					Baseline HbA1c% = 7.58 ± 1.7 10 weeks = 7.38 ± 1.5	
Vaisman *et al.*, 2009 [28]	Randomized Controlled Trial	13 12	79.2 ± 10.4 (Mean ± SD) 73.0 ± 14.7 (Mean ± SD)	Standard tube feed Diabetes Specific Tube feed	HbA1c = 7.9 ± 0.3% at baseline, 8.7 ± 0.4% at 12 weeks	Results are expressed as mean ± SEM
					HbA1c = 6.9 ± 0.3% at baseline, 6.2 ± 0.4% at 12 weeks	
Alish CJ *et al.*, 2010 [27]	Randomized, double blinded crossover design (Postprandial glycaemic and Insulinemic response)	22	63.1 ± 1.9	DSFs STFs	Baseline plasma Glucose = 113.1 ± 6.9 mg/dL	Results are expressed as mean ± SEM
					Adjusted (change from baseline) plasma glucose conc. = 22.3 ± 4.4 mg/dL	
					Baseline insulin levels = 21.2 ± 2.9 μL/mL	
					Peak insulin levels = 79.5 ± 17.2 μL/mL	
					Baseline plasma Glucose = 124.8 ± 5.3 mg/dL	
					Adjusted (change from baseline) plasma glucose conc. = 71.1 ± 7.0 mg/dL	
					Baseline insulin levels = 16.3 ± 1.7 μL/mL	
					Peak insulin levels = 115.2 ± 28.0 μL/mL	

With respect to the study by Vaisman *et al.* [28], HbA1c decreased over time in the diabetes specific diet group and increased in the standard feed group. Similarly, in the randomised double-blinded study evaluating the postprandial glycaemic and insulinemic response conducted by Alish *et al.* [27], differences in adjusted peak plasma glucose levels were significantly lower in DSF compared with STF (*p* < 0.001). In addition, differences in adjusted peak insulin levels were significantly lower in DSF compared with STF (*p* = 0.017). In other words, the use of DSF produced significantly lower postprandial and insulinemic response.

Voss [29] revealed that adjusted glucose concentrations were significantly higher at all points after feeding the STF compared with the SDC or DSF (*p* < 0.001). In addition, adjusted plasma glucose concentrations for the SDC were observed to be significantly lower than those for the DSF from 30 to 120 min (*p* < 0.05). In relation to the adjusted insulin responses, these were significantly higher for the STF compared with the SDC at each time point, while the SDC had a significantly lower insulinemic response compared with the DSF at 90 and 120 min (*p* < 0.05) [29]. Finally, the adjusted results for postprandial GLP-1 levels at 30 and 60 min in this study were significantly lower for the STF compared with SDC (*p* < 0.05), but not different from the DSF [29].

In the study by De Luis *et al.* [30] which compared diabetes specific enteral supplement with 49.95% calories provided by fat (Gp 1) and diabetes specific enteral supplement with 34% calories provided by fat (Gp 2), a significant decrease of glucose and HbA1c was observed in Gp 1 compared with Gp 2.

## 4. Discussion

Diabetes specific formula seems to be effective in managing glucose profiles including postprandial glucose and insulinemic response and HbA1c compared with standard formula in patients with diabetes on enteral nutrition. The effectiveness of DSF in patients with diabetes on enteral feed may be partly due to the form of carbohydrate used in its formulation. Diabetes specific formulas often contain carbohydrate that are more slowly digested and absorbed compared with standard formula that contain carbohydrates that are more rapidly digested and absorbed [27]. Postprandial blood glucose response, which is a risk factor for micro- and macro-vascular complications, has been shown to be influenced profoundly by the specific composition of the diet [31]. The measure of how soon glucose reaches the blood stream is often termed the glycaemic index (GI) of food while the glycaemic load (GL) shows the overall glycaemic effect of a specific amount of food item [1]. It is possible that DSFs are formulated with carbohydrates which have lower GI compared with standard formulas. In a study conducted by Hofman *et al.* [32], it was shown that the GI of the 12 enteral formulas determined in the study varied widely from GI = 12 for a diabetes specific feed up to GI = 61 for a standard supplement. High GI foods have GI value ≥70 while medium GI values range from 55 to 70 compared to low GI foods that have GI value ≤55 [33]. According to Hofman *et al.* [32], in general, a low GI formula (DSF) is characterised by reduced carbohydrate content, presence of fructose, a higher fat content containing monounsaturated fatty acids (MUFA) and high amounts of fibre while standard formulas, especially sip feeds, often do not contain fibre. This view is reinforced by Charney and Hertzler [34] who noted that diets containing up to 30% of total calories as MUFA have led to improvements in lipoprotein levels and glycaemic control in patients with diabetes. In addition, small doses of fructose (5 g to 10 g) have been found to be effective in reducing acute glycaemic response to a carbohydrate challenge partly because of the low GI of fructose, which is 19 compared with the GI of glucose which is 100 [34]. On the other hand, standard enteral formulas, whether oral or enteral, are high in carbohydrate, low in fat and fibres compared with DSFs which contain defined nutrient composition such as fructose, fibre, MUFA, soy bean and antioxidants which are designed to improve glycaemic control [19]. The source of carbohydrate in DSF includes increased amount of fructose relative to standard formulas and fat often in the form of higher amounts MUFA when compared with standard formulas although issues around tolerance on high levels of fats and fructose in feed have been subjects of discussion [22,35]. The main sources of fibre in DSF are usually fruits and vegetables and the levels are relatively higher than in standard formulas.

Evidence of a meta-analaysis demonstrates that low GI foods have clinically more useful effect on medium-term glycaemic control (glycated proteins) in patients with diabetes [32]. According to Widanagamage *et al.* [36], the long-term use of foods that have high GI can place a higher metabolic demand on the body in terms of higher insulin requirement with the potential to lead to insulin resistance.

## 5. Conclusions

The outcomes of the studies that compared Diabetes specific formula with standard formulas appear to show a trend with respect to the response of glucose and other parameters such as HbA1c, which define diabetes. Based on the evidence in the present review, there are indications that the use of Diabetes specific formula could be effective in managing glucose in patients with diabetes on enteral nutrition.
